# A case of pleomorphic dermal sarcoma^؆^

**DOI:** 10.1016/j.abd.2026.501318

**Published:** 2026-03-23

**Authors:** Yin Lv, Xudong Mou, Yuelin Li, Jian Li, Hong Ren, Renqiong Chen

**Affiliations:** a Lianyungang Clinical College, Nanjing Medical University, Lianyungang, Jiangsu, China; b Jinzhou Medical University, Jinzhou, Liaoning, China; c Department of Dermatology, The Affiliated Lianyungang Hospital, Xuzhou Medical University, Lianyungang, Jiangsu, China; d Department of Dermatology, The First Affiliated Hospital of Kangda College, Nanjing Medical University, Lianyungang, Jiangsu, China

Dear Editor,

Pleomorphic Dermal Sarcoma (PDS) is a rare cutaneous mesenchymal neoplasm that shares a clinicopathologic spectrum with Atypical Fibroxanthoma (AFX).^1^, ^2^, ^3^ PDS predominantly occurs in sun-damaged areas of elderly patients, with a male predilection (M:F = 7:1).^1^ Clinically, it typically presents as a rapidly growing solitary nodule, often with ulceration and bleeding.^1^, ^2^, ^3^ We herein report a case of PDS involving the face of an elderly female patient.

A 95-year-old woman presented with a left cheek mass that developed two months following minor facial trauma. The lesion progressed rapidly, with recurrent ulceration and bleeding. Her medical history was unremarkable for chronic conditions.

Physical examination showed a 3 × 3.5 cm pink, exophytic nodule with surface hemorrhagic crust and no tenderness on palpation (Fig. 1). Histopathological examination revealed deep dermal infiltration by pleomorphic spindle cells and multinucleated giant cells, with areas of necrosis and hemorrhage. The tumor cells showed focal storiform patterning, high mitotic activity and atypical forms (Fig. 2). Immunohistochemistry was positive for CD10 and CD68 but negative for CK, desmin, CD34, p63, S100, and SOX10 (Fig. 3). These findings led us to confirm the diagnosis of PDS.

**Fig. 1:**
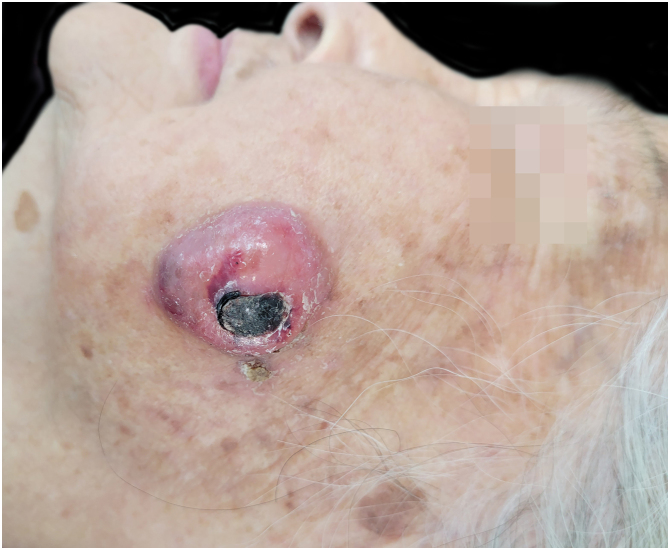
A red exophytic nodule approximately 3 × 3.5 cm on the left cheek of the patient. precisa girar esta imagem 90 graus esquerda.

**Fig. 2:**
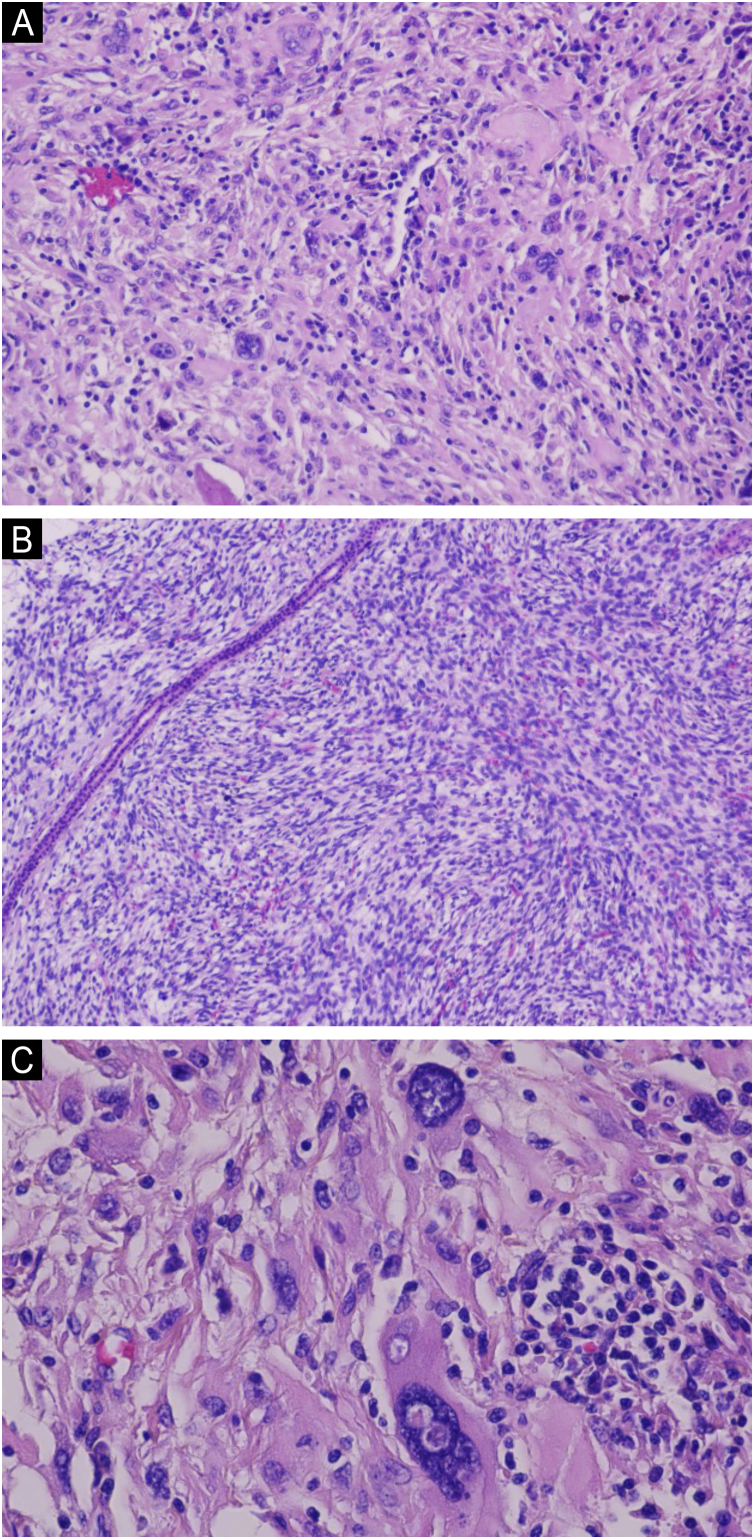
(A) Neoplastic architecture demonstrating tumoral infiltration within the dermis, composed of pleomorphic spindle cells along with multinucleated giant cells (Hematoxylin & eosin, ×200). (B) Some tumor cells are arranged in a storiform pattern (Hematoxylin & eosin, ×200). (C) Demonstrating very atypical cells (Hematoxylin & eosin, ×400).

**Fig. 3:**
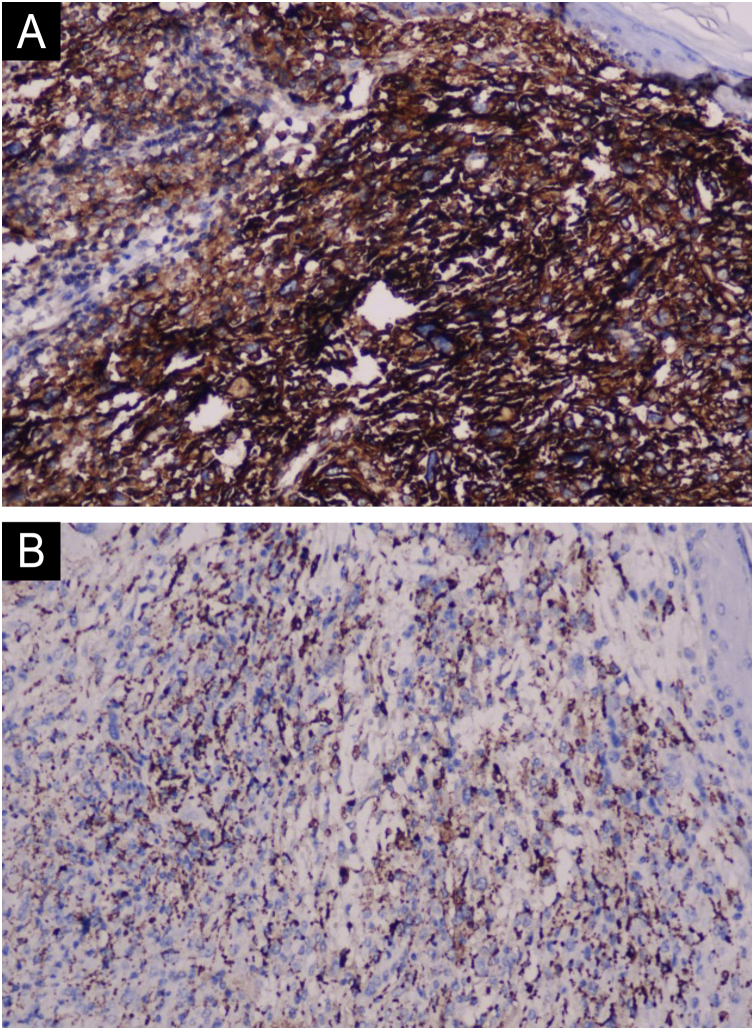
(A) Positive staining for CD10. (B) CD68 (Hematoxylin & eosin, ×200).

PDS is a rare cutaneous neoplasm of purported mesenchymal differentiation that exists along a clinicopathologic spectrum with AFX.^1^, ^2^, ^3^ Historically, AFX was referred to as ‘superficial Malignant Fibrous Histiocytoma (MFH).^2^ With the advent of contemporary histologic techniques, many tumors previously deemed as MFH have been reclassified into specific pathologic entities, while the remaining unclassifiable sarcomas were designated as ‘Undifferentiated Pleomorphic Sarcoma’(UPS).^1^, ^2^, ^3^, ^4^, ^5^, ^6^ However, UPS encompasses a heterogeneous group of malignant soft tissue tumors, including some deep soft tissue tumors. Hence, the term PDS was proposed to refer specifically to cutaneous neoplasms involving the dermis and subcutaneous tissue.^1^, ^2^, ^3^

PDS predominantly affects sun-exposed areas in elderly patients, particularly the head and neck region, with a male predilection. Tumors typically present as a rapidly growing solitary nodule that may ulcerate and bleed.^1^, ^2^, ^3^, ^4^, ^5^ Histologically, PDS is composed of numerous pleomorphic spindle cells and epithelioid cells, with the presence of multinucleated giant cells. Tumors are non-encapsulated with ill-defined borders. Neoplastic cells possess abundant cytoplasm, hyperchromatic and irregular nuclei and atypical mitoses are often present.^1^, ^2^, ^3^, ^4^, ^5^ Diagnosis of PDS requires a systematic immunohistochemical work-up, as there are currently no specific IHC markers for this entity. While CD10 positivity is observed in most cases, this marker lacks specificity and cannot confirm the diagnosis.^1^, ^2^, ^3^, ^4^ The differential diagnosis between PDS and AFX is crucial, as their prognoses differ significantly. AFX is a relatively low-grade cutaneous neoplasm with rare metastatic potential. In contrast, PDS is associated with a higher risk of both local recurrence and distant metastasis. PDS exhibits more aggressive histopathologic features, including invasion into the deep dermis or subcutaneous tissue, lymphovascular invasion, and/or perineural invasion. Compared to AFX, PDS is usually larger, more asymmetric, and less well-circumscribed. Most PDS cases demonstrate tumor necrosis, crusting, and hemorrhage.^1^ A comprehensive cytokeratin panel should be obtained to exclude spindle cell squamous cell carcinoma. Melanocytic markers, such as S100, HMB-45, and Melan-A must be evaluated to rule out spindle cell or desmoplastic melanoma.^1^, ^2^, ^3^, ^4^ It should be noted that focal S100-positive dendritic cells may be present in PDS, and multinucleated giant cells may demonstrate Melan-A immunoreactivity.^1^, ^4^ The tumor cells were negative for desmin, which could distinguish it from leiomyosarcoma. Some cases of PDS may may demonstrate CD31 positivity, However, CD34 and ERG are consistently negative in PDS, which is helpful for the differential diagnosis of angiosarcoma.^1^, ^4^

At present, there is no standard for the management of PDS.^4^ Surgical resection remains the gold standard of treatment and usually leads to a cure.^1^, ^2^, ^3^, ^4^, ^5^ Positive or borderline surgical margins are the main risk factors for recurrence, so it is generally recommended to use a wide resection range of at least 1 cm.^4^ The role of adjuvant radiotherapy is not clear, and it is suitable for high-risk features such as nerve and vascular invasion, but the tolerance of elderly patients needs to be balanced. Chemotherapy and radiotherapy can be used as salvage treatments for recurrence and distant metastasis.^1^, ^2^, ^3^, ^4^, ^5^ The prognostic factors for PDS remain incompletely understood, as the TNM staging system does not apply to this tumor type. Current evidence identifies advanced age, distant metastasis, and larger tumor size as independent risk factors associated with poorer outcomes.^4^, ^6^

In our case, the patient developed a facial tumor following minor trauma, which rapidly enlarged over a period of two months. The tumor was relatively large, measuring 3 × 3.5 cm. Although there was no evidence of lymphovascular or perineural invasion, histopathological examination revealed deep dermal infiltration, with some areas showing storiform arrangement, numerous atypical mitotic figures, along with tumor necrosis and hemorrhage. Based on these findings, we ultimately made the diagnosis of PDS. The patient underwent surgical excision, and microscopic examination showed negative surgical margins. The patient has been followed up for two years postoperatively, with no evidence of tumor recurrence or metastasis to date.

## ORCID IDs

Yin Lv: 0009-0007-0726-9343

Xudong Mou: 0009-0000-8718-2924

Yuelin Li: 0009-0003-0379-7246

Jian Li: 0009-0007-1504-358X

Hong Ren: 0000-0003-3156-6640

Renqiong Chen: 0000-0002-0625-316X

## Authors' contributions

Yin Lv: Study concept and planning; data collection, analysis and interpretation; critical literature review; preparation and writing of the manuscript; approval of the final version of the manuscript.

Xudong Mou: Study concept and planning; data collection, analysis and interpretation; critical literature review; preparation and writing of the manuscript; approval of the final version of the manuscript.

Yuelin Li: Study concept and planning; Intellectual participation in propaedeutic and/or therapeutic management of studied cases; critical literature review; approval of the final version of the manuscript.

Jian Li: Study concept and planning; intellectual participation in propaedeutic and/or therapeutic management of studied cases; critical literature review; approval of the final version of the manuscript.

Hong Ren: Study concept and planning; intellectual participation in propaedeutic and/or therapeutic management of studied cases; critical literature review; approval of the final version of the manuscript.

Renqiong Chen: Study concept and planning; intellectual participation in propaedeutic and/or therapeutic management of studied cases; critical review of the literature; approval of the final version of the manuscript.

## Financial support

This study was supported by grants from the Science Foundation and Research and Development Fund Project (XYFZ202305) and Lianyungang 521 Project (LYG065212024075).

## Research data availability

Does not apply.

## Conflicts of interest

None.
